# OASIS evaluation of the French laboratory diagnostic surveillance system: right people, right techniques but imperfect use

**DOI:** 10.3389/fvets.2025.1419034

**Published:** 2025-09-16

**Authors:** Maïssane Chikh, David Ngwa-Mbot, Eric Morignat, Sophie Memeteau, Jean-Philippe Amat

**Affiliations:** ^1^Laboratory of Lyon, Epidemiology and Surveillance Support Unit, University of Lyon—French Agency for Food, Environmental and Occupational Health & Safety (ANSES), Lyon, France; ^2^National Federation of Farmers’ Animal Health Services (GDS France), Paris, France; ^3^French Health and Environmental Association, Paris, France

**Keywords:** animal health, surveillance, laboratory diagnostic tests, OASIS evaluation, veterinary medicine, assessment

## Abstract

Laboratory diagnostic surveillance is the surveillance of incidents and the risk of incidents, resulting from the use of diagnostic tests. The role of this surveillance is to detect the potential mistakes in laboratories’ analytic methods and defects in diagnostic tests. We assessed the diagnostic surveillance system dedicated to five cattle diseases in France: infectious bovine rhinotracheitis (IBR), brucellosis, hypodermosis, bovine viral diarrhea (BVD) and enzootic bovine leukosis, using OASIS, a method developed for the assessment of surveillance systems. Information regarding the organization and functioning was collected during semi-structured interviews with actors taking part in the laboratory diagnostic surveillance system, including staff at national reference laboratories, diagnosis laboratories, veterinarians, diagnostic test manufacturers, cattle owners’ association and veterinary services. A scoring grid of 78 criteria was completed, based on the insights collected during the interviews. This scoring was then used for the calculation of seven surveillance critical control points based on the hazard analysis of critical control points approach and of ten quality attributes of the system. Key performance factors included: good technical management of laboratories, a monitoring of the performance of diagnostic tests by laboratories (intern control charts) and a good level of expertise for all actors. The areas of improvement were related to the lack of formalized bodies (steering committee, scientific and technical committee, coordinators, etc.), the lack of reporting guidelines, insufficient feedback to actors (regarding incidents and functioning of the system), and the absence of a definition of a case in laboratory diagnostic surveillance. In order to address these flaws, we recommend a new organization. Other main proposals for improvement included: establishing guidelines for reporting and investigating; raising the awareness of the actors concerning laboratory diagnostic surveillance; and establishing feedback meetings focused on the events of laboratory diagnostic surveillance. Such an evaluation should be conducted for other diseases and in other countries. It would be useful to share the results, especially within Europe, to implement improvements at the European level.

## 1 Introduction

In Europe, the animal health law (1) classifies diseases in different categories of importance and regulates their surveillance. According to the World Organization for Animal Health (WOAH), surveillance refers to the systematic, ongoing collection, collation, and analysis of information related to animal health, and the timely dissemination of information so that action can be taken (2). This definition emphasizes the importance of continuous monitoring and a quick response to maintain animal health and prevent the spread of diseases. Surveillance implies the use of laboratory analysis and diagnostic tests for the detection of cases, including reagents. A reagent is a test substance “added to a system in order to bring about a reaction or to see whether a reaction occurs” (3).

In France, the performance of most diagnostic tests and reagents is assessed by national reference laboratories (NRL) before being used by field diagnostic laboratories. After this initial evaluation step, the performance of tests is assessed routinely throughout the monitoring of the laboratory’s results. These laboratory results are shared with the official administrators of the animal disease surveillance and can lead to suspect defects associated with diagnostic tests. Depending on the disease, the official administrators (both private and public) of disease surveillance may be either the local veterinarian services or the local cattle owners’ association, known as GDS in French departments and FRGDS in French regions.

We defined “laboratory diagnostic surveillance” (LDS) as the surveillance of incidents and risk of incidents resulting from the use of diagnostic tests and reagents. Although this type of surveillance does not monitor a direct threat to animal health, the definition of animal health surveillance can be transposed to such laboratory diagnostic surveillance because it involves the ongoing collection, collation, and analysis of information related to diagnostic test incidents or suspicion of diagnostic test incidents, and the timely dissemination of information so that action can be taken with the need for a continuous monitoring and quick response. All the actors involved in this surveillance and their interactions constitute the LDS system. The role of this surveillance is to detect the potential defects in analytic methods in laboratories and diagnostic tests. In this context, any disruption within the diagnostic chain could significantly impact the surveillance and control of animal diseases. Consequences of incidents associated with diagnostic tests may include: (i) a decrease in specificity, that would result in an increase in the number of animals erroneously identified as positive, potentially triggering false epidemic alerts. This could result in the loss of the “infection-free” status for specific herds and, in extreme cases, necessitate the preemptive culling of healthy animals. (ii) Conversely, a decrease in sensitivity would result in animals being incorrectly classified as negative and could lead to the spread of the diseases within and between the herds and incur economic losses for animal owners (4). Such misdiagnoses might lead to delays in detecting epidemic outbreaks, exacerbating the spread and severity of disease. Each incident detected by the system may be due either to a diagnostic test failure or to another reason, in particular a true significant change in the epidemiological situation of the disease, such as an increase or a decrease in the number or cases or outbreaks. LDS actors have to investigate each incident to determine its origin. In this way, the LDS can also inform and contribute to disease surveillance.

In this context, the assessment of the quality and the efficacy of the LDS system is necessary to verify that the current organization and operation of the system enables the quick and comprehensive detection of incidents and their management, and suggest possible improvements. The current LDS system in France involves different stakeholders that are represented nationally (e.g., NRLs of the diseases) and locally in the French departments (GDSs, local veterinary services and field laboratories). Interactions between these different actors can vary across departments, depending on the willingness of each to contribute to the LDS. This variability in the strength of these interactions may constitute a significant source of variability in surveillance quality. Thus, the objective of this study was to assess the operation and organization of the LDS system for five cattle diseases in France, in order to identify its strengths and weaknesses and to propose potential improvements. This evaluation was conducted with the OASIS evaluation tool (5) based on the feedback of stakeholders of the surveillance system.

## 2 Materials and methods

### 2.1 The laboratory diagnostic surveillance (LDS) system

The French LDS system is composed of two components. The first one can be qualified as “event based surveillance,” and covers all diagnostic tests used by state-approved laboratories. It is based on the declaration of any incident or suspected incident resulting from a defect of the characteristics or performance of a diagnostic test. All users of diagnostic tests (e.g., manufacturers, field laboratories technicians) must declare any suspicion of abnormality concerning the tests to the NRLs, as described in the French regulation (6). The incidents in laboratory test results can be a signal for LDS and/or disease outbreaks detection. LDS focuses on defects in the tests, such as their sensitivity and specificity. It concerns all animal diseases with official surveillance. The LDS system is composed of two levels: the national level and the local level. The national level includes NRLs, the French General Directory for Food (DGAL), the National Federation of Farmers (GDS France, blood and milk laboratory representatives associations and diagnostic and reagent tests manufacturers). NRLs are responsible for granting marketing authorization of reagents. They are also in charge of scientific and technical support to laboratories and the investigation of all incidents notified by other actors. Through the DGAL, the Ministry of Agriculture is in charge of the regulation of LDS and of the surveillance of certain animal diseases. The laboratories representatives associations federate laboratories approved for analyses on blood and milk, separately. Diagnostic test and reagent manufacturers also take part in LDS, as they produce and sell the tests after they are approved by the NRL. The local level is composed of the administrators of the diseases, local veterinary services, and local associations of cattle owners, namely GDS in departments and FRGDS in regions. At the local level, LDS field actors also include veterinarians and laboratories, as they, respectively, collect and analyze the samples; the latter are direct users of diagnostic tests.

The second component of LDS system is an active surveillance based on the use of control charts mutualized by laboratories belonging to an association of diagnostic laboratories, named ADILVA. This component tracks the use of reagents alongside a standard reference material, through their routine application by certain laboratories represented by ADILVA. The active surveillance component is currently implemented for infectious bovine rhinotracheitis (IBR) and Johne’s disease (Gibout, personal communication).

Since the active surveillance component is exclusively implemented in a few laboratories and limited to only two diseases, we focused our evaluation on the first component, which provides comprehensive national coverage and includes all diseases without exception.

### 2.2 Evaluation method

The LDS system was assessed with the OASIS method. The OASIS method (5) is regularly used to assess surveillance systems in different countries in animal health, and more recently in plant health and food safety (7–10). This method is standardized and results in a detailed assessment of the system of interest.

### 2.3 Selected diseases

In this study, we focused on five ruminant diseases: infectious bovine rhinotracheitis (IBR), bovine viral diarrhea (BVD), brucellosis of cattle and small ruminants, bovine hypodermosis and enzootic bovine leukosis. The choice of these diseases was based on different criteria: (1) the existence of an NRL for the disease, (2) the existence of a national disease eradication plan, (3) the diversity of diagnostic methods (ELISA, PCR) and matrixes (blood, milk, biopsies), (4) the existence of more than one manufacturer of diagnostic tests and reagents and (5) the diversity of susceptible species: cattle, sheep, goat. For all those diseases, active surveillance is implemented and consists in annual prophylaxes for which diagnostic tests are used. IBR is known worldwide to cause significant economic losses to the cattle industry. Control programs were initially established in the 1970s, but were reinforced by the implementation of requirements for IBR-free status, by the European Union (EU), as a prerequisite for the importation of cattle, semen and embryos. This regulatory framework has prompted intensified eradication efforts among EU member states (11). Several European nations and regions have successfully achieved IBR-free status, including Austria, Finland, Norway, Switzerland and specific territories of Italy (12). However, control strategies across Europe exhibit considerable heterogeneity and are largely nation-specific. France and other countries have not achieved IBR-free status but maintain official control campaigns characterized by annual prophylactic measures and vaccination protocol (11, 12).

BVD is another disease that inflicts substantial economic losses on the cattle industry globally (13). Effective vaccines have been developed and demonstrate efficacy in preventing vertical transmission to offspring and horizontal transmission within herds (14, 15). Alternative non-vaccination strategies, based on screening and certification, have also proven successful, as it is the case in Scandinavian countries (16, 17). An approach based on direct testing of all new-born calves for viral antigen has been developed in Switzerland and then applied in several countries (18). Approaches combining vaccination with targeted eradication of seropositive cattle have been adopted in several European nations, including Germany, Ireland and France (17, 19, 20). Each approach has specific advantages depending on regional prevalence, industry structure and regulatory framework.

Enzootic bovine leukosis imposes economic burden through production losses and health intervention costs, while also increasing host susceptibility to secondary infections in affected animals (21, 22). In order to facilitate international trade, the EU nations have implemented official control programs for bovine leukosis (23). Unlike other major bovine viral diseases, no effective vaccine currently exists for it (24). Control strategies primarily comprise surveillance protocols incorporating systematic screening of cattle herds, with subsequent movement restrictions applied to those testing positive (21, 25–28). The global distribution of bovine leucosis exhibits distinct regional patterns; within the EU, the disease is restricted to limited areas including Germany, France, Greece and Portugal, while 19 EU nations have obtained the free-leukosis status by the EU (29).

Bovine hypodermosis compromises animal welfare through the formation of subcutaneous nodules and associated stress response. This disease also has economic consequences on milk and beef production, and costs associated with treatment intervention (30, 31). Considering the economic benefits of the eradication of bovine hypodermosis, several nations have implemented eradication programs, which generally incorporate systematic treatment protocols, movement restrictions and surveillance. Despite these coordinated efforts, the disease continues to persist in several countries (30–32).

Brucellosis ranks among the most prevalent zoonotic diseases globally, requiring robust control measures to mitigate its impact on both animal health and human health (33). Several nations have successfully achieved brucellosis-free status through eradication programs, including several EU nations, Canada, Australia, New-Zealand and the United Kingdom (34). The success in these regions can be attributed to systematic test-and-slaughter policies, vaccination programs, movement restrictions, and rigorous surveillance protocols maintained over extended periods. In contrast, brucellosis remains endemic across substantial portions of the globe, including Western Asia, the Indian subcontinent, Near East countries, and numerous South American nations (34, 35).

### 2.4 Diagnostic tests

Official disease surveillance in France involves annual screening, with samples analyzed in laboratories that have been validated by the Ministry of Agriculture. For brucellosis, IBR, enzootic bovine leukosis and bovine hypodermosis, analyses can be performed on both serum and bulk milk samples using enzyme-linked immunosorbent assay (ELISA) techniques, and other techniques are also used such as virus isolation, immunohistochemistry and PCR.

For IBR detection, indirect ELISA demonstrates high efficiency for bovine herpesvirus 1 (BoHV-1) antibody detection in blood, even pooled, and milk samples. More specifically, whole-virus indirect ELISA is suitable for bulk milk testing, while blocking ELISA are commonly used for confirming bulk milk positive results and for beef farms, especially gB ELISA that shows specificity and sensitivity higher than 99% (12, 36–39).

For BVD surveillance, virus neutralization test has long been considered the gold standard, but ELISA employed for cattle serum is now the primary method since it provides a simple, fast method, with high sensitivity and specificity and well suited to handling large numbers. However, such methods based on antibody detection fail to identify persistently infected animals, unlike antigen or virus testing such as virus isolation, reverse transcription polymerase chain reaction (RT-PCR), immunohistochemistry or antigen capture ELISA (13, 40–43).

For bovine leukosis detection, ELISA methodology effectively identifies antibodies in both serum and bulk milk, with good specificity and sensitivity comparing with agar gel precipitation (AGID) or phytohemagglutinin (PHA) tests (44–47). Quantitative PCR may also be used for proviral load, after ELISA screening, to help to prioritize the most infectious cattle for segregation or culling (21).

Regarding hypodermosis surveillance, ELISA is commonly used for serum and milk samples mainly due to its high specificity and sensitivity, although these parameters vary according to the type of ELISA test: indirect, competitive, sandwich, etc. (32, 48–50).

For brucellosis, multiple analytical methods are employed. While bacteriological analysis remains the gold standard (51, 52), ELISA is widely used for detection in serum and bulk milk samples (53–56). Additional methodologies for antibody detection include rose bengal test (RBT) (53, 55, 57), serum agglutination test (SAT) (55, 58), and complement fixation (CF) (56, 57, 59, 60) which has previously served as a confirmatory test following RBT. ELISA demonstrates superior operational simplicity while generally maintaining high sensitivity and specificity, although these vary depending on the manufacturer: some ELISAs, but not all, outperform other serological methods (60, 61). Despite these advantages, ELISA performance may be affected by vaccination status and disease prevalence in certain contexts. Since the different tests have not perfect sensitivity and specificity, it is usually recommended to combine several tests for brucellosis surveillance (61–63). In France, several kinds of tests, including ELISA, CF, buffered antigen plate agglutination test, ring test, and bacteriology, are routinely used within the surveillance program (56).

### 2.5 OASIS evaluation process

The first step of the OASIS assessment consists of semi-directed interviews of several actors involved in the system at national and local levels (5), led by an assessment team composed of internal assessors (involved in the system, generally members of the coordination team) and external assessors (epidemiologists not involved in the system) (Table 1). For our evaluation protocol, we implemented a multi-level sampling approach to capture relevant perspectives across the LDS system hierarchy. At the national level, we recruited representatives from the DGAL involved in animal health regulation, NRL coordinators for each disease, technical experts from GDS France and representatives from laboratory associations for blood and milk analyses. At the subnational level, we selected personnel from local GDS offices, including veterinarians, technicians, and administrative leaders. Additionally, we selected laboratory technicians and directors from laboratories that analyze both blood and milk samples. Veterinarians responsible for field sample collection within the surveillance program were also selected. To ensure representativeness of interviewed actors while maintaining feasibility, the evaluation team selected representatives from each professional category, strategically distributed across different geographical regions. Our regional selection criteria were multifactorial, considering: (i) diversity in ruminant species distribution (cattle, sheep and goat); (ii) the spectrum of laboratory diagnostic techniques employed on the different matrixes (blood and milk); (iii) heterogeneous prevalence patterns of IBR and BVD, which present variable distribution across France (unlike the three other diseases under surveillance, which are absent or nearly absent nationally). This methodological approach was designed to effectively capture the heterogeneity of epidemiological situations and operational contexts present throughout the LDS system.

**Table 1:** Composition of the evaluation team for the OASIS assessment of the French LDS system.

Name	Function and institute	Internal or external assessor
Sophie Memeteau	Veterinary—Animal technical division of the French health and environmental association	Internal
Jean-Philippe Amat	Head of the Unit Epidemiology and surveillance support at ANSES	External
Maïssane Chikh	Junior epidemiologist—PhD student at ANSES and GDS France	External

This approach resulted in the identification of 30 organizations, including national and subnational representatives, for a total of 23 semi-directive interviews conducted between June and November 2021. Interview sessions included one or two representatives from each participating organization (Table 2). The interviews generally lasted an hour and a half and the different fields that compose a surveillance system were discussed. Those ten fields or topics (“functional sections”) were the objectives and scopes of the surveillance, the central institutional organization, the field institutional organization, the practices of the laboratories, the surveillance tools used in the system, the surveillance procedures, the data management, the training of the actors involved in the system, the communication, and the evaluation and the performance indicators.

**Table 2:** Actors interviewed for the OASIS evaluation of the French LDS system.

Category of actor	Representative chosen for the evaluation	Role of the interlocutor interviewed
DGAL^a^	Animal health bureau	In charge of animal diseases regulation and surveillance
	Laboratories bureau	In charge of LDS regulation
GDS France^b^	Leucosis/Brucellosis	National technical representative
	BVD/IBR	
	Hypodermosis	
NRL^c^		NRL in Niort:
	NRL for IBR/ Leucosis	Laboratory manager
	NRL for Hypodermosis	Laboratory manager
	NRL for BVD	Laboratory manager
		NRL in Maisons-Alfort:
	NRL for Brucellosis	Person in charge of the reference+ technical manager
GDS/FRGDS^d^	FRGDS Bretagne	Person in charge of health surveillance
	FRGDS Bourgogne—Franche-Comté	Director
	GDS of Aude	Director
	GDS of Saône-et-Loire	Director
	GDS of Maine-et-Loire	Director
Local veterinary services	Local veterinary services of Rhône	Head of service + deputy head
Diagnostic laboratories	Laboratory of Charente	Director
	Laboratory of Aveyron	Director
	Laboratory of Doubs	Director
	Laboratory of Nord	Director
	Laboratory of Maine-et-Loire	Director
	Laboratory of Puy-de-Dôme	Director
	Laboratory of Finistère	Director+ technical manager
ADILVA		Person in charge of the control card scheme + President + Vice-President
Veterinarian practitioner		Veterinarian (Doubs)
		Veterinarian (Charente)
		Veterinarian (Maine-et-Loire)
Diagnostic kit manufacturer		Marketing manager + person in charge of laboratory technical support

a DGAL: French General Directory for Food.

b GDS France: National Federation of Farmers.

c NRL: National reference laboratory.

d GDS/FRGDS: Local cattle owner associations.

The second step consisted of filling a notation grid of 78 criteria (5), with the information collected during the interviews and in documents related to the system, such as regulations, procedures, or agreements. Each criterion was marked from zero (lowest grade) to three (highest grade) based on the system’s performance, according to a detailed scoring guide (5). The grid and the scoring guide were adapted to the context of the LDS system, as it differs from a usual epidemiological surveillance system dedicated to diseases that the OASIS system was originally designed for. These adaptations consisted in adapting the vocabulary of certain criteria and adapting the scoring guide; for instance, the “case” was not defined as an animal/human/plant infected but as an incident or a suspicion of incident in a laboratory test result (Supplementary material 1). Following the interviews, the grid was pre-filled by the evaluation team and then reviewed during a one-day meeting by a” notation team” composed of the evaluation team and representatives of stakeholders of the LDS system: national reference laboratory, veterinary services, cattle owners association, diagnostic laboratories, veterinarian practitioner, diagnostic tests manufacturers and experts of reagent control (Table 3). The results of the scoring are illustrated by three figures, automatically generated by the OASIS tool: pie charts of the scoring of each functional section, a histogram of seven critical control points (objectives, sampling, coordination, tools, data collection, data analysis and interpretation, information distribution) and a radar chart of ten attributes (sensitivity, specificity, flexibility, timeliness, representativeness, stability, acceptability, simplicity, usefulness, reliability).

**Table 3:** Composition of the notation team for the OASIS evaluation of the French LDS system.

Institute	Function
ANSES^a^ – GDS France^b^	Junior epidemiologist (member of the evaluation team)
ANSES^a^	Head of the unit Epidemiology and surveillance support (member of the evaluation team)
French health and environmental association	Animal health expert
GDS France^b^	Veterinary—Animal technical division of the French health and environmental association (member of the evaluation team)
DGAL^c^	Official veterinary of the Animal health Bureau
ANSES^a^	Head of the NRL IBR
ANSES^a^	Head of the unit pathology and animal welfare (in charge of IBR, BVD, enzootic bovine leucosis and hypodermosis NRL)
ANSES^a^	Leader of the working group on reagents control
Laboratory of analyses/representative of ADILVA^d^	Laboratory director/ member of ADILVA
National society of veterinarian practitioners	Veterinarian practitioner
Union of veterinary reagent and medicinal products manufacturers	General secretary
FRGDS^e^ Bourgogne – Franche-Comté	Director

a ANSES: French Agency for Food, Environmental and Occupational Health & Safety.

b GDS France: National Federation of Farmers.

c DGAL: French General Directory for Food.

d ADILVA: Association of representatives of diagnostic laboratories.

e FRGDS: Local cattle owner associations.

Thirdly, based on the grid, the figures, and the supporting comments completed by the notation team, the evaluation team wrote a report on the state of the system and proposed recommendations for improvement.

### 2.6 Ethical approval

The OASIS evaluation of the French laboratory diagnostic surveillance system did not require ethical approval as we collected information through interviews with actors participating in the system. However, we informed participants that all information collected during the interviews would be anonymized and used for the study and publication. All of them gave their agreement.

## 3 Results

**Figure 1:**
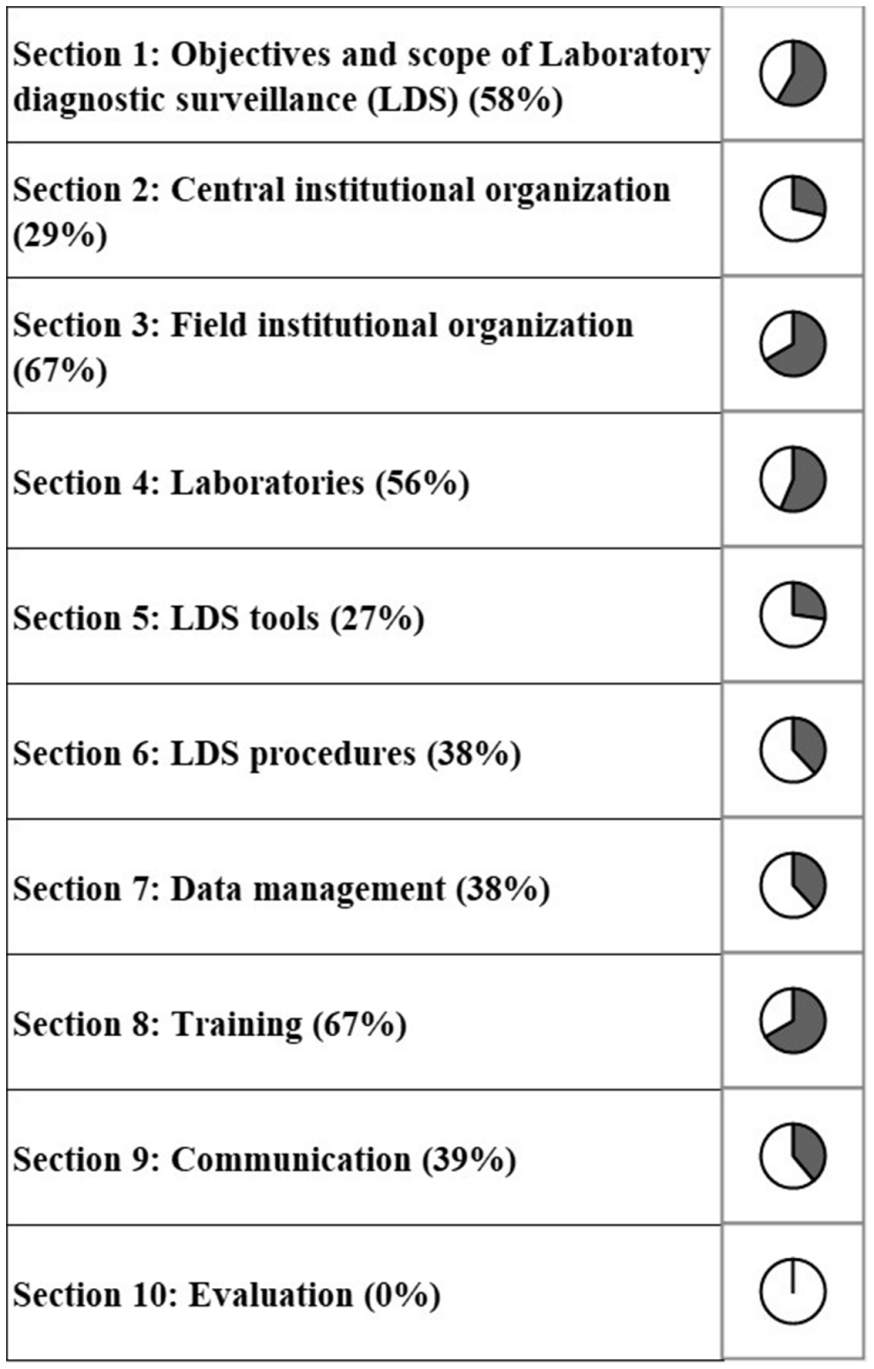
Scores of the French laboratory diagnostic surveillance (LDS) system for infectious bovine rhinotracheitis (IBR), bovine viral diarrhea (BVD), enzootic bovine leucosis (EBL), hypodermosis and brucellosis obtained for the 10 OASIS functional sections (the score of each section is displayed as a percentage score).

**Figure 2:**
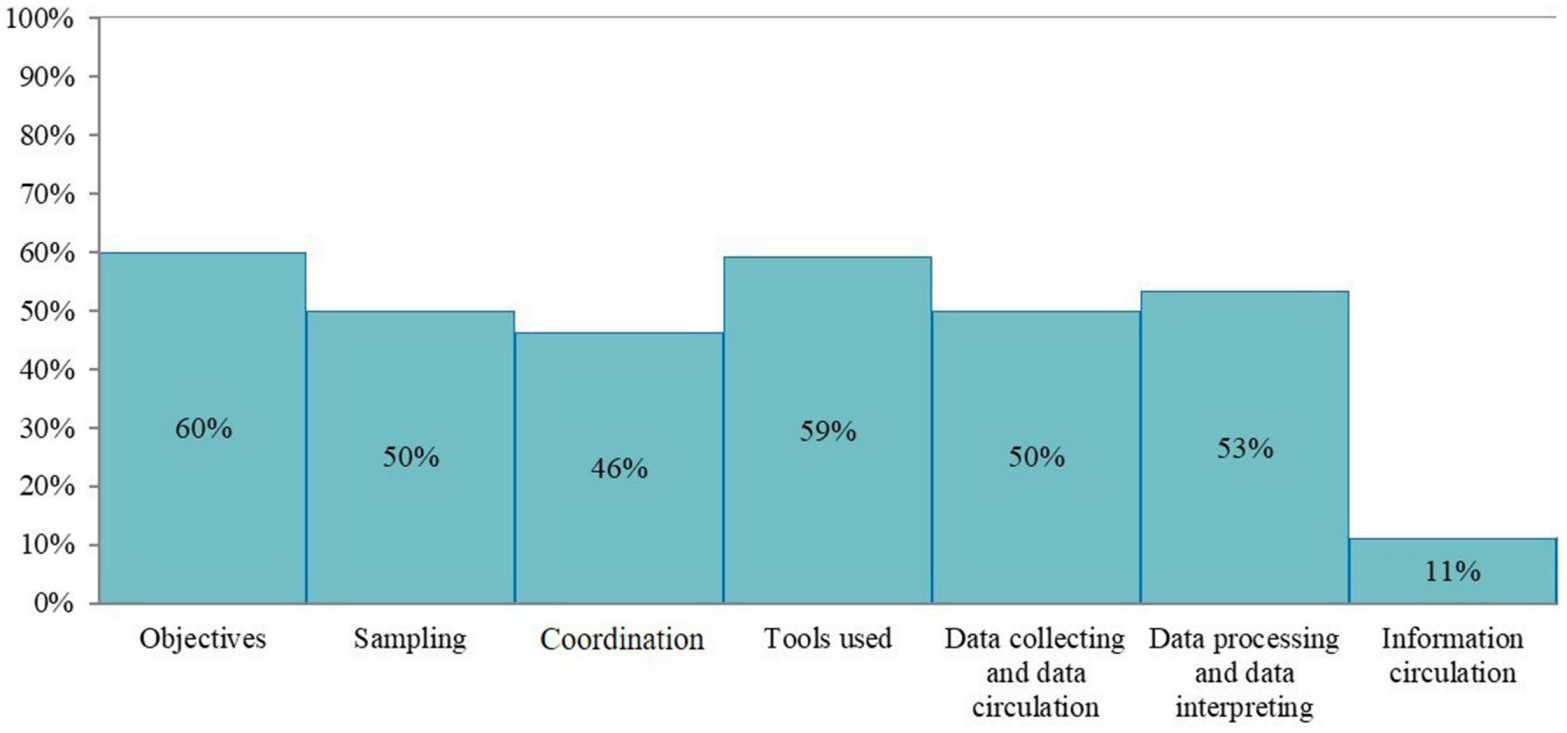
Scores of the French laboratory diagnostic surveillance (LDS) system for infectious bovine rhinotracheitis (IBR), bovine viral diarrhea (BVD), enzootic bovine leucosis (EBL), hypodermosis and brucellosis obtained for the seven OASIS critical control points (the level of satisfaction for each critical control point is displayed as a percentage score).

**Figure 3:**
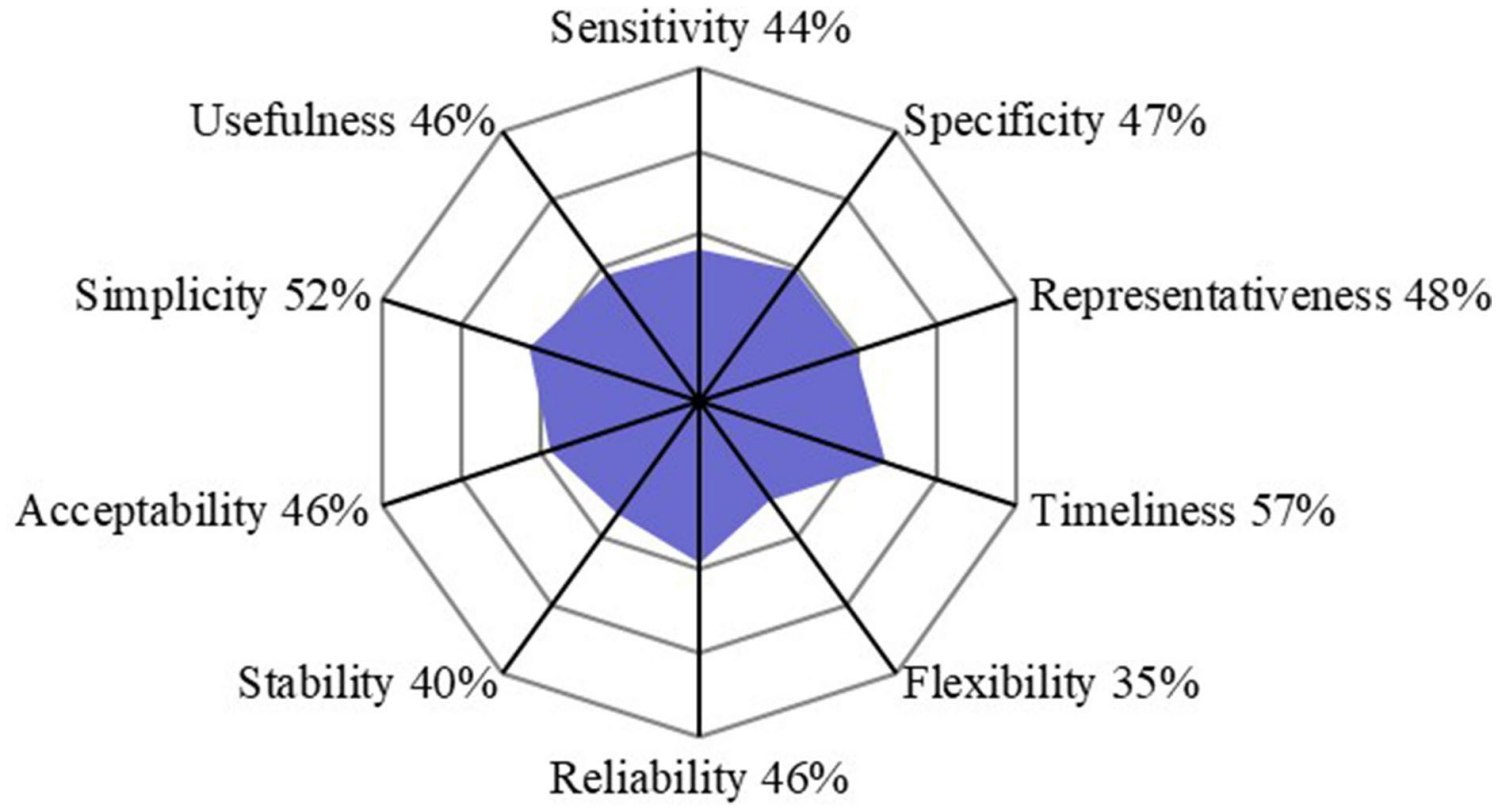
Scores of the French laboratory diagnostic surveillance (LDS) system for infectious bovine rhinotracheitis (IBR), bovine viral diarrhea (BVD), enzootic bovine leucosis (EBL), hypodermosis and brucellosis obtained for ten OASIS attributes (the level of satisfaction for each attribute is displayed as a percentage score).

The completed scoring grid was used for the calculation of scores that are shown in the three outputs of the evaluation (Figures 1–3). The highest scores were obtained for the field institutional organization (67%) and training (67%) among functional sections (Figure 1), objectives (60%) and tools (59%) for critical points (Figure 2), timeliness (57%) and simplicity (52%) for attributes (Figure 3). No score was above 67%. The lowest scores were obtained for the evaluation (0%, Figure 1), information circulation for critical points (11%, Figure 2), and flexibility for attributes (35%, Figure 3). The results of the evaluation were reviewed by functional section in light of the attributes and critical points.

### 3.1 Objectives

All the actors that were interviewed considered the objectives of the LDS system relevant, although they are not formalized in any form. This affects the usefulness (46%) and stability (40%) attributes (Figure 3). For improvement, formalizing the objectives and making them easily accessible to all stakeholders was recommended (Table 4).

**Table 4:** Main recommendations for improvement of the French laboratory diagnostic surveillance (LDS) system for infectious bovine rhinotracheitis, bovine viral diarrhea, enzootic bovine leucosis, hypodermosis and brucellosis regarding the ten OASIS functional sections.

Functional section of the LDS system	Main recommendations
1. Objectives and scope of LDS	Easy access to the formalized objectives.
2. Central institutional organization	Establish a steering committee, a scientific and technical committee and coordinators for the LDS system.
3. Field institutional organization	Establish an intermediate unit that would ensure the relay between the central unit and the field actors.
4. Laboratories	Educate the field actors on the importance of alerting more systematically the NRLs in case of a defect in the diagnostic tests so they can support the investigations.
5. LDS tools	Standardize a protocol of declaration of incident, stating whom to contact, when, and with which kind of information.
6. LDS procedures	Establish surveillance methods for incident detection and management that accommodate better to the sake of LDS.
7. Data management	Define interesting data to follow for LDS purposes. Create a laboratory-generalized database for the LDS events, accessible by NRLs.
8. Training	Reinforce the training, especially continuous training, of certain field actors (veterinarians, local veterinarian services)
9. Communication	Regularly discuss the LDS incidents and functioning during formal meetings at national and local levels to improve the feedback and operations. A LDS protocol should improve the communication.
10. Evaluation	Following the better formalization of the LDS system, define and calculate performance indicators to follow and improve the performance of the system.

### 3.2 Central institutional organization

The LDS system lacks a formalized central institutional organization, resulting in decreasing scores of the associated section (29%, Figure 1) and of the coordination critical point (Figure 2). It also affects the stability, acceptability and flexibility attributes (Figure 3). While the DGAL should lead the steering committee, there is no such committee, nor a scientific and technical committee, or coordinators for the system, all of which usually constitute the central institutional organization of a surveillance system. In practice the NRLs take on most of the responsibility for activities of the central unit and scientific and technical committee. The DGAL is responsible for the regulation of laboratory activities, including LDS, but it has very few exchanges with other actors regarding LDS and it has not defined the organization and operations of the LDS system. Moreover, a steering committee should be composed of all the parties involved in the system and meet regularly to make the main decisions, which is not the case currently.

A scientific and technical committee usually gathers all technical skills of interest to produce and keep updated a surveillance protocol and to define the surveillance modalities, from data collection to data analysis and communication. The main actors dedicated to such activities in the LDS system are GDS France, laboratory associations, veterinarians, diagnostic tests manufacturers, veterinary services and above all NRLs. However, the absence of a body gathering all those actors concerned by this topic prevents them from providing collective scientific and technical support as needed to ensure relevant surveillance.

NRLs partly act as a central unit (i.e., coordination team) thanks to their expertise and relationships with all actors. Nevertheless, they are not clearly in charge of coordinating field actors, centralizing and analyzing the results, coordinating trainings and being a relay in communication, which usually are key missions for a central unit within a surveillance system. In the case of a diagnostic tests defect suspicion, diagnostic laboratories can request the NRLs for their expertise, but it does not happen systematically and quickly (sometimes only several weeks after identification of a defect). Since NRLs are not informed of all the suspicions, they cannot centralize all the data concerning the incidents of diagnostic tests nor investigate all of them.

Regarding communication, annual meetings such as the Professional Reference Days and the NRL Days (gathering diagnostic laboratories, GDS, local veterinary services and manufacturers) provide an opportunity to keep the professionals involved in the LDS system and updated on what happened during the latest or on-going surveillance campaign. However, the incidents related to diagnostic tests and the results of the LDS system are not always discussed during these meetings, though major events are usually addressed.

The different actors involved in the LDS system generally have important knowledge of the history of the system and good technical and scientific expertise. Formalizing the different committees and defining their composition and missions would improve the system, its stability and flexibility (i.e., adaptation to the health or regulatory context for instance), and it would allow for a better use of the knowledge and expertise of the actors.

### 3.3 Field institutional organization

Field actors cover all the country. Even if their missions regarding LDS are not formalized, they carry out most of the activities needed, including identification of the suspicions of incidents, which contributes to the reliability of the system (Figure 3) and to the good score of this section (67%, Figure 1). GDSs supervise the sample collection and veterinarians collect the samples on animals. The diagnostic laboratories analyze the samples, use the diagnostic tests and reagents, and control the quality of the results. The veterinarians and the official administrators for the diseases (GDSs and local veterinarian services) generally know the historical and health status of farms well, which is very useful to interpret test results (especially unexpected results), in collaboration with laboratories. Local coordination of actors and data analysis is mostly done by GDSs, more particularly for IBR, BVD and hypodermosis. The official administrators are present in all French departments and can be alerted by diagnostic laboratories or veterinarians if they have suspicions regarding the validity of the analyses results. All these field actors collaborate for investigations in the case of an incident, even by involving the diagnostic tests manufacturer and some colleagues from neighboring departments if needed. However, they do not always inform the NRL at this stage. If no solution is found between the field actors, the NRL is generally alerted thereafter. Therefore, at the local stage, communication is usually fast. At the national stage, communication with NRLs is not efficient, as they are not often informed of the investigations of defects. This is the cause of the relatively low timeliness score (53%, Figure 3) and of the coordination of the system (46%, Figure 2). The communication between the central organization (NRLs, DGAL) and field actors is limited, not only in case of incidents but also for sharing general information. The establishment of an intermediary body that would ensure the relay between field and central actors would harmonize their collaboration by improving data circulation (Figure 2).

Moreover, the communication between the local official administrators and the diagnostic laboratories concerning test incidents is variable depending on the departments and can be influenced by multiple factors such as the prevalence of the disease in the department, the geographical proximity of these actors or the quality of their relationship. Coordination meetings at the local level, focusing on the main livestock diseases, exist but they focus mainly on epidemiological surveillance, not on LDS, and their frequency depends on the department. They can take place multiple times during the annual prophylaxis or only once a year for the contract review between GDS, veterinarians and laboratories, which seems insufficient to treat all issues. Different topics are discussed during those meetings, including sometimes the diagnostic test incidents that happened.

### 3.4 Laboratories in the LDS system

Diagnostic laboratories play an important role in the system, as they are the users of the diagnostic tests and usually the first to detect a possible incident regarding them. The laboratories’ technical expertise, quality management systems, and detection methods including internal control charts and control markers, contribute to the relatively high performance, compared to the other sections, observed in laboratory functional section (56%, Figure 1) and the critical control point related to tools (59%, Figure 2), despite the insufficient development of resources specifically designed for LDS. Laboratory technicians leverage their technical knowledge to rapidly investigate potential system deficiencies, i.e., unexpected results, initially through internal review processes and subsequently through collaborative approaches with external partners (including other diagnostic laboratories) when issues exceed local resolution capacity. For example, when a laboratory encounters an unusual spike in positive results for a specific disease, technicians first verify internal quality controls to rule out laboratory contamination or an issue with reagents before contacting other regional laboratories to check if similar patterns have been observed, or contacting diagnostic test or reagent manufacturers. In case of detection of systematic deviations in control charts for a specific disease, the laboratories usually first check testing equipment, reagent quality and procedures and only contact the NRL in the event of persistent inconsistency. Indeed, there is no procedure explaining who and how to alert in case of an incident, so the actors involved in the investigations (official administrator, laboratories, NRL, veterinarians) depend on the department. This lack of standardization in the investigations negatively affects the reliability of the LDS system (46%, Figure 3).

All actors recognize NRLs as experts on the diagnosis tools and their support would be useful to investigate anomalies. However, in practice, NRLs are often informed only if no solution can be found with the field actors or the manufacturer, sometimes several weeks after the beginning of the issue. The system could gain efficacy and timeliness from a standardized protocol stating the actors to contact in case of suspicion (Table 4). Moreover, a centralization of all incidents at the NRL level would allow a more global surveillance of diagnostic tests that are used throughout the prophylaxis campaign. Indeed, informing a national actor such as the NRL may help to quickly identify, investigate and fix a problem occurring in several departments, which is not always possible when anomalies are only treated at the local level for several weeks or months.

### 3.5 Tools and procedures of LDS

In the LDS system, there are no formalized procedures for incident registration and investigation. In addition, there is no clear definition of an incident in the LDS context that is approved by all the stakeholders. The lack of such procedures and definition dedicated to LDS impacts the functional sections related to the tools and procedures (27 and 38% respectively, Figure 1). In the field, an investigation is very dependent on the health situation and the relationship between local actors in the department, introducing heterogeneity. Moreover, the lack of definition for a LDS case affects negatively the simplicity of the system (52%, Figure 3), given that the decision of alerting is not straightforward for field actors. The lack of procedures implies that there is no defined delay for registration and data circulation and no pre-conceived format for data collecting and processing (Figure 2). This also decreases the score of representativeness (48%, Figure 3). Some laboratories developed their own notification sheets for incidents and they can send it to NRLs. However, these sheets are not harmonized among laboratories and not systematically used.

In addition to the current surveillance component based on the detection by field actors of incidents through the analysis of diagnostics results, a second surveillance component, still under development, will be based on national control charts managed by ADILVA. This second approach should allow rapid data reporting. However, it will likely concern only a small number of diseases and not all the laboratories only ADILVA members.

The event-based surveillance is implemented on the whole territory but the lack of a formalized framework for the surveillance causes heterogeneity in the participation of the local actors that impacts the sampling (50%, Figure 2), the representativeness and the sensitivity (48 and 44% respectively, Figure 3). Laboratories monitor diagnostic results with internal control charts, in the framework of their accreditation and their quality systems. They can detect quickly a defect and report the information to manufacturers and/or official administrators. The regular exchanges between laboratories and other field actors and the good knowledge of the epidemiological situation of the farms by the official administrators and veterinarians allow the investigation of incidents and their reporting to the central level, namely the NRL. However, this reporting to NRL is neither systematic nor harmonized: some field actors are more used to informing the NRL than others, depending on their experience and willingness. There are actions to raise the awareness of field actors to the detection and reporting of incidents, via network days.

The two components, event-based and programmed, appear to be complementary. However, the LDS system as a whole would be improved by a more homogenous notification of incidents and from a national control chart tool generalized to all laboratories. The results of this latter tool should be consultable by NRLs, and even by GDS France, laboratories and manufacturers for certain data, depending on their level of confidentiality, to increase the sharing of information and the collective interest for LDS.

### 3.6 Data management

The FRGDS and the diagnostic laboratories have their own databases to manage the analysis of diseases surveillance and LDS data and to identify possible incidents, in connection with their knowledge of epidemiological data and the implementation of control tools in the laboratories (control charts). Thanks to their databases, the FRGDS follow alerts from the declaration to the resolution. Each laboratory holds a register of non-conformities, which also acts as a database for recording and tracking anomalies associated with diagnostic tests. These registers include information on the description of incidents, the analysis of causes and impacts and the measures taken. In the same way as for the FRGDS, these databases are specific to each laboratory, they are not interconnected and do not feed a national database. More specifically, there is no dedicated national database for LDS, preventing from a comprehensive data analysis. It also affects the scoring of reliability (46%, Figure 3) and the scoring of the two critical control points related to data collection, processing and interpretation (50 and 53% respectively, Figure 2). The implementation of a centralized database and the adequate analysis of its data would need human and technical resources, currently insufficient at a national level.

Regarding the programmed surveillance component, ADILVA has its own database for the management of national control chart data. The NRLs do not yet have access to this database, but it is planned in the short term.

### 3.7 Training

Regarding the central unit (NRLs), the skills and knowledge in analytical reference are very satisfactory and those in epidemiology are globally solid, increasing the scoring of reliability (Figure 3). The staff of the diagnostic laboratories and of the FRGDS follow training courses when joining the laboratory, and thus the LDS system; for the FRGDS, this is systematic for certain diseases within the framework of their quality accreditations. Such initial training courses do not focus on LDS but include it in their program. However, experience and knowledge of the health history of the cattle population are irreplaceable in acquiring solid skills and being able to detect an anomaly in the context of LRV. The training, especially continuous training, of certain actors (veterinarians, local veterinary services) should be reinforced.

### 3.8 Communication

Results of incident investigations by the NRLs are communicated to the alerting actors. A minority of actors from diagnostic laboratories regret that some NRLs are not available to respond to their requests, while the vast majority are satisfied with the level of exchange. The feedback to veterinarians should be improved, especially when they have been requested during investigation.

A written report of the incidents related to LDS is not made or published for either the public or even the actors involved in the system, decreasing the scoring of the information dissemination critical control point (11%, Figure 2). Such a regular report would be particularly useful to laboratories, (FR)GDS and manufacturers, especially if the NRLs were systematically informed of anomalies detected in the territory. However, the NRLs and GDS France, as well as the annual meeting of the NRLs and their networks of diagnostic laboratories already report a certain number of investigations of anomalies at different meetings: the National Professional Reference Day that is organized annually.

There are more local meetings, departmental or regional, with variable frequencies (one to several per year) in which news related to LDS is sometimes discussed. Such meetings gather official administrators, veterinarians’ representatives and laboratories, but dairy laboratories are sometimes absent or not represented.

Routine communication between the different actors of the system by e-mail or phone is generally adequate but lacks formalization, especially in the case of alert. A shared platform for incident declaration could benefit the system by allowing the access to a unique and common notification tool to all field actors.

### 3.9 Evaluation

This study was the first evaluation of LDS system in France. Moreover, as the system is not yet formally organized, the operating indicators have not yet been put in place (Table 4), which explains why this section received a score of 0% (Figure 1).

### 3.10 Recommendations for the LDS system

**Figure 4:**
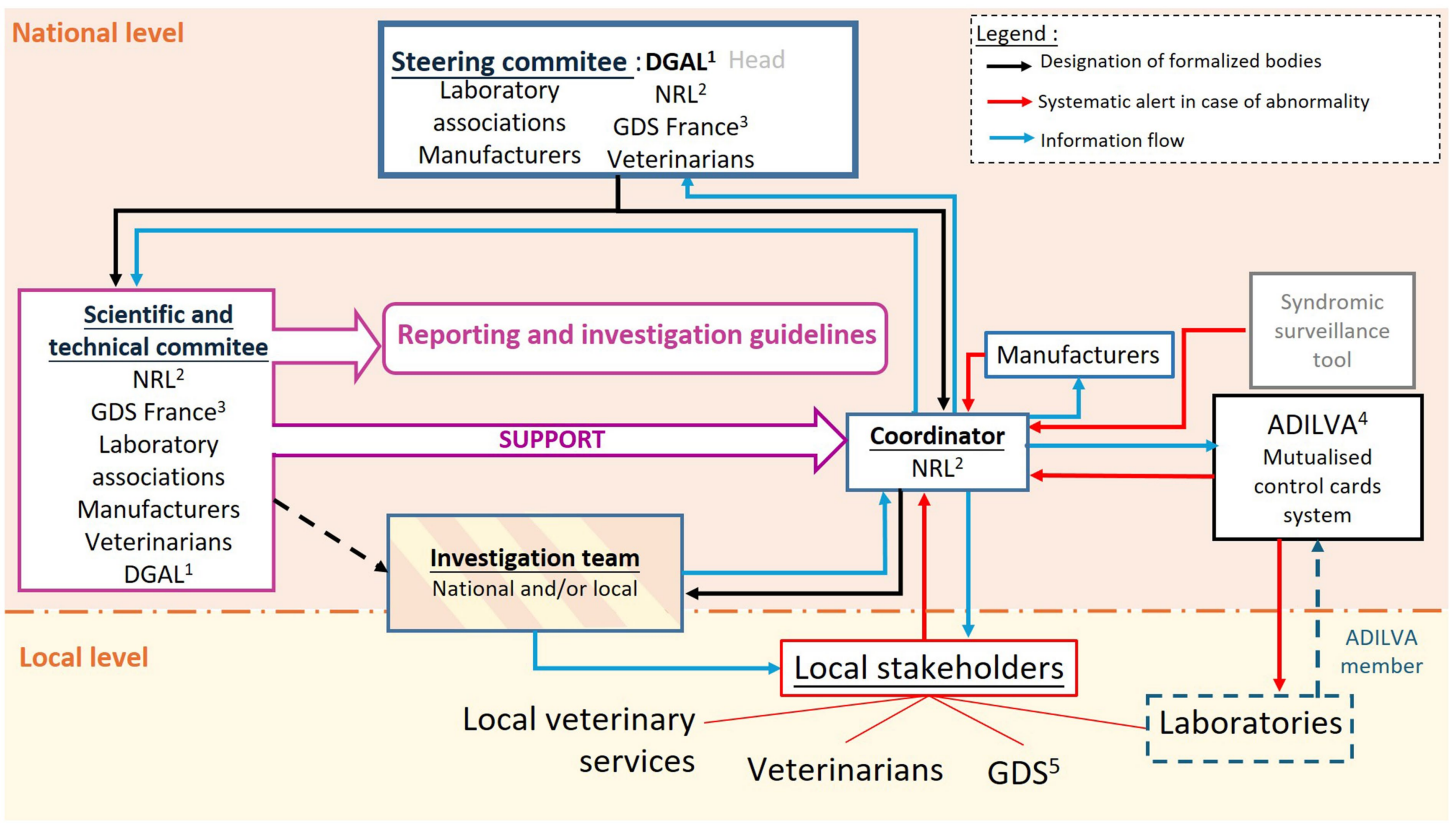
Proposed organization of the French laboratory diagnostic surveillance (LDS) for animal health, considering the recommendations made through the OASIS evaluation. ^1^DGAL: French General Directory for Food; ^2^NRL: National reference laboratory; ^3^GDS France: National Federation of Farmers; ^4^ADILVA: Association of representatives of diagnostic laboratories; ^7^GDS: Local cattle owner associations.

Based on the results of the evaluation, we propose a new organization of the LDS system with defined committees that have formalized missions (Figure 4). This organization would be common to multiple diseases such as IBR, brucellosis, leucosis, hypodermosis and BVD. A steering committee would be responsible for defining the organization and key directions of the surveillance system (Figure 4), including the establishment of necessary entities, their functions, and the formalization of their functions. This committee would meet regularly during the system formalization phase but less frequently thereafter, ideally aiming for an annual meeting.

A scientific and technical committee, common to all animal diseases included in the LDS system, would bring together technical and scientific expert stakeholders such as the NRLs, GDS France, laboratory associations, national representatives of manufacturers, and veterinarians (Figure 4). Collaborating within this committee would enhance the functioning and results of the LDS system. This committee would be tasked with developing guidelines for incident recognition and management, from reporting to resolution, as well as overseeing investigations.

The staff of NRLs would play a key role as system coordinators, centralizing data in case of suspicion, designating an investigation team when necessary, and disseminating alerts and updates on investigations. Additional human resources would probably be needed for well ensuring this mission. Moreover, a research topic is currently being developed to investigate the contribution of an automatic incident detection tool. Based on syndromic surveillance using prophylaxis data collected nationally, this tool would complement the LDS system and would aim to detect diagnostic test defects in near real-time. It would inform coordinators of the LDS system with automated alarms. The coordinators should then identify whether the alarm is due to a diagnostic test defect or a change in the number of cases.

An investigation team, designated according to the guidelines of the scientific and technical committee, would be responsible for probing suspicions (Figure 4). Comprising members selected based on their expertise, both at the local and national levels, this team would regularly report to the coordinators and to local stakeholders. Financial and technical resources for these investigations could come from a dedicated national fund or, alternatively, the organizations of the members of the investigation team, though this funding raises questions of acceptability and limitations regarding the frequency of investigations.

## 4 Discussion

### 4.1 Strengths and weaknesses of the LDS French system

To our knowledge, it is the first time that the French animal health LDS system is assessed. This evaluation provides an overview of the organization of the system by pointing out its strengths and weaknesses and suggesting recommendations.

According to this evaluation, one of the main strengths of the French LDS system is the presence of field actors that are qualified but not properly educated on LDS. In fact, they practice LDS but implicitly in the context of Laboratory certification. The expertise of NRLs is also an asset to investigate and resolve incidents. Another strength of the system is the existence of tools such as control charts (for each laboratory and, hopefully soon, shared) and a national database for disease surveillance data, named SIGAL, but they are not exploited for the purpose of LDS. Internal control charts and data from SIGAL can be used in case of an investigation, following an alarm, but the investigations are not registered. However, the degree of usage of data depends on the department. The complementary tool based on syndromic surveillance would support the actual LDS system, especially in terms of timeliness and coverage of analytical results to monitor.

Part of the weaknesses of the system come from the lack of a formalized definition of an LDS “case,” i.e., an anomaly or suspicion of anomaly in diagnostic results potentially linked to a defect of the diagnostic tests. A definition would allow for a better education of the actors who would detect more efficiently LDS cases. However, if such a definition does not yet exist, it is probably because defining an anomaly could be tricky: it depends on many epidemiological and analytical factors, which could be interpreted differently for each herd. The confirmation of a case relies on several analyses, carried out on pooled samples and then on individual samples, the definition of an anomaly could be even more complicated. Typical problems that concern LDS are: (i) results that do not match the clinical presentation of the animals or known disease prevalence in the region; (ii) unusually high positive or negative rates from a specific batch of diagnostic tests compared to historical data; (iii) multiple herds showing similar unexpected results when tested with the same reagent or diagnostic test; (iv) pooled samples that test positive but subsequent individual samples from the same animals test negative. Many more problems could also be taken into account in a future definition, based on the feedback of field actors and other experts.

Some results of the evaluation were not expected. Among critical control points and attributes, apart from the Objectives that has a score of 60%, the best scores were obtained for Tools (59%, Figure 2) and Timeliness (57%, Figure 3). The score for Tools considered a good integration of laboratories in disease surveillance systems, with good diagnostic techniques, which are highly necessary for LDS but not specific to it. Financial and material resources, deemed sufficient given the current state of the system, influenced positively the score for Timeliness. However, LDS incidents are underestimated, and the resources would be probably insufficient to carry out quickly all investigations if all abnormalities were detected and reported. The formalization of a LDS, as described in Figure 4 would need significantly more financial and human resources than are allocated for the current system. However, this cost would decrease as the system begins to run routinely. It is also important to notice that the Timeliness score is not really high, reflecting that, on one hand, the field actors investigate suspicions of abnormalities as fast as possible and, on the other hand, the NRLs are often informed only several weeks after the beginning of the incident.

Overall, almost all the scores were relatively close and neither function, critical control point nor attribute were above 67%, illustrating that there is stillroom for improvement. Increasing the formalization of central bodies and protocols would be a priority; this will require exchanges between all stakeholders and collaborative constructions, which would automatically improve many other features of the LDS system. We did not find any study previously conducted and published on LDS in any other country, for any livestock sector, preventing the comparison with others LDS systems.

### 4.2 OASIS evaluation tool

The OASIS tool was originally created for the evaluation of epidemiological surveillance systems (5). In the context of LDS, the wording of certain criteria and scoring guides needed some modifications. The adaptations established by the evaluation team included a definition of a “case” in the field of LDS (i.e., an incident or a suspicion of incident in a laboratory test results), and the target population was not animals, but the diagnostic tests used by the laboratories. In addition, evaluation of criteria related to the data were focused on the LDS data, not on disease surveillance data. The adaptations were understood and validated by the notation team and allowed for a smooth review of the grid. However, the adaptation of criteria for the Laboratory section was not easy considering that these criteria were originally mainly focused on the diagnostic methods of the laboratories and there is no specific method in laboratories for LDS. These criteria were then focused on the activities, resources and tools dedicated to the detection of a diagnostic test anomaly, rather than to the detection of a pathogen, as usually done within an OASIS evaluation. Additionally, criteria related to the quality, reliability and standardization of samples were considered as irrelevant, as there are no samples to detect a diagnostic tests defect.

Interviews with DGAL and a few local veterinary services were initially considered for the evaluation. However, we could not interview either of them during the initial period of the evaluation. The reasons for their absence can be diverse but the one systematically given was their lack of time. It does not seem that the results of the evaluation were significantly affected by the lack of information coming from the DGAL, considered the authority of the system, given that the DGAL is not actively involved in the LDS system for incident detection and investigation. For local veterinary services, we could arrange an interview with delegates of one department, later than expected but before the notation meeting. This allowed us to take into consideration their experience, even though it cannot be generalized to all the other departments since the level of awareness and implication of the local actors may depend on the local prevalence and the history of epidemic outbreak. The feedback of many other field actors (laboratories, GDS, veterinarians) about local veterinary services activities was useful to partially mitigate this unique interview with local veterinary services.

The OASIS tool is based on the feedback of actors involved in the system, collected during semi-directive interviews, which can imply subjectivity. However, the composition of the evaluation team (intern member who knows the system and extern members who are newbies to the system), the detailed scoring guide and the scoring team, composed of delegates of the stakeholders of the system, permit to lessen the subjectivity.

### 4.3 Recommendations

All the main recommendations for the LDS system (Figure 4) ask for financial and human resources, at least working time, that should be estimated with the system stakeholders, as they are the best to estimate the associated costs. The formalization of the system, counting the establishment of the instances, the elaboration of the procedures and the formalization of a clear definition of a LDS case, if possible, would certainly need resources, but they are of the utmost importance to significantly improve the surveillance. Moreover, one of the main expected benefits of improving the LDS system is to avoid delays in the detection of outbreaks and false positive results for herds, leading to cost savings for the animal industries, official administrators and competent authorities. Even though confirmation test is usually performed by the NRL, it is not the case for all diseases and the IBR example presented in this article showed that the confirmatory precautions were not always sufficient to prevent the false positive diagnosis (12).

Thus, the increase in the costs due to the improvement of the LDS system could improve the health status of herds and secure livestock trade and should be considered as an investment. Once the new system is in place, we could expect a decrease in the cost, as it would run routinely, although the cost associated with investigations will depend on the number of defects in the diagnostic test detected. The complementary system, based on syndromic surveillance, will have a higher cost as it would need to be developed from scratch (64). In fact, the implementation of this system will be time consuming and will need dedicated people, as for other syndromic surveillance systems (5). However, the cost of this system may be reduced overtime with standardization of the tool (8).

## 5 Conclusion

Overall, the French LDS system for five cattle and small ruminants’ infectious diseases is composed of competent actors that possess valuable knowledge. However, the lack of formalization and communication affects negatively the efficiency of the system. The proposed recommendations aim primarily at improving the formalization of the central institutional bodies and the surveillance protocols. It would be useful to assess the cost-effectiveness of such measures in the future, considering the animal health and economic issues. Such improvements of LDS would disease management by laboratories and NRLs. Evaluating the efficiency of the LDS for other diseases and livestock productions would be useful to improve the system at the national level. Carrying out such evaluations in other countries would allow us to better understand the implementation of such systems and make it possible to share recommendations.

## Data Availability

The original contributions presented in the study are included in the article/Supplementary material, further inquiries can be directed to the corresponding author.
